# Crystal structure of caspase recruiting domain (CARD) of apoptosis repressor with CARD (ARC) and its implication in inhibition of apoptosis

**DOI:** 10.1038/srep09847

**Published:** 2015-06-03

**Authors:** Tae-ho Jang, Seong Hyun Kim, Jae-Hee Jeong, Sunghwan Kim, Yeun Gil Kim, Hyun Ho Park

**Affiliations:** 1 Department of Biochemistry, Yeungnam University, Gyeongsan, 712-749, South Korea; 2 Pohang Accelerator Laboratory, Pohang University of Science and Technology, Pohang, 790-784, South Korea; 3 New Drug Development Center, Daegu-Gyungpook Medical Innovation Foundation, Daegu, 701-310, South Korea

## Abstract

Apoptosis repressor with caspase recruiting domain (ARC) is a multifunctional inhibitor of apoptosis that is unusually over-expressed or activated in various cancers and in the state of the pulmonary hypertension. Therefore, ARC might be an optimal target for therapeutic intervention. Human ARC is composed of two distinct domains, N-terminal caspase recruiting domain (CARD) and C-terminal P/E (proline and glutamic acid) rich domain. ARC inhibits the extrinsic apoptosis pathway by interfering with DISC formation. ARC CARD directly interacts with the death domains (DDs) of Fas and FADD, as well as with the death effector domains (DEDs) of procaspase-8. Here, we report the first crystal structure of the CARD domain of ARC at a resolution of 2.4 Å. Our structure was a dimer with novel homo-dimerization interfaces that might be critical to its inhibitory function. Interestingly, ARC did not exhibit a typical death domain fold. The sixth helix (H6), which was detected at the typical death domain fold, was not detected in the structure of ARC, indicating that H6 may be dispensable for the function of the death domain superfamily.

Cell death and proliferation must be balanced for multi-cellular organisms during developmental stages, as well as for homeostasis, and cells that present a threat to the integrity of an organism have to be removed. Apoptosis is a well-known mechanism for elimination of cells that maintains tissue homeostasis and protects organisms from accumulation of dangerous cells^1^,^2^. Failure to control apoptosis leads to serious diseases, including various cancers^3^,^4^. The hallmarks of apoptotic cells are cell shrinkage, cytoplasmic and nuclear condensation, chromatin condensation, and ordered DNA fragmentation^5^,^6^. An intrinsic (mitochondrial) and an extrinsic (cytoplasmic) pathway lead to apoptosis^7^,^8^. The extrinsic pathway is triggered through death receptors, which are members of the tumor necrosis factor (TNF) receptor superfamily, whereas the intrinsic pathway leads to the release of cytochrome-c from the mitochondria, thereby activating the death signal^8^. Both extrinsic and intrinsic pathways ultimately converge, leading to activation of a cascade of cysteine proteases known as caspases, which are a family of cysteine proteases that cleave specifically after aspartic acid residues^9^,^10^. Caspases are divided into two classes based on their roles in apoptosis, initiator caspases such as caspase-2, -8, -9, and -10, and effector caspases such as caspases-3 and -7. Initiator caspases are auto-processed upon induction of dimerization by recruitment to oligomeric signaling complexes^11^. Well-known oligomeric signaling complexes for the activation of initiator caspases include the death-inducing signaling complex (DISC) for caspase-8 activation^12^, the apoptosome for caspase-9 activation^13^, and the PIDDosome for caspase-2 activation^14^,^15^. Effector caspases are activated upon cleavage by activated initiator caspases^10^,^16^.

BCL-2 family proteins such as BAX, BAK, BCL-2, BCL-XL, BCL-w, and BCL-B are required for regulation of the intrinsic cell death pathway^17^. These proteins can trigger both pro-apoptotic (BAX, BAK, BAD and PUMA) and anti-apoptotic (BCL-2, BCL-XL, BCL-w, and BCL-B) pathways. Members of the pro-apoptotic family, BAX and BAK, form pores on the mitochondrial membrane through which apoptotic signals such as cytochrome-c and Smac are released into the cytosol, where they help to activate effector caspases for execution of apoptosis^18^.

Apoptosis repressor with caspase recruiting domain (ARC) is a multifunctional modulator of apoptosis. ARC is predominantly expressed in post-mitotic cells such as cardiomyocytes, neurons, and muscle cells, and unusually over-expressed in various cancers^19^,^20^. Involvement of ARC in cancer development is not surprising because over-activated anti-apoptotic proteins can render cells insensitive to apoptotic signals. Over-activation of ARC has also been detected in pulmonary hypertension^21^. Additionally, it has been reported that hypo-activation of ARC causes fulminant liver failure, which is rescued by treatment with ARC protein^22^. ARC has also recently been reported to be involved in TNFα-induced necroptosis^23^. Taken together, the results of these previous studies suggest that ARC is critical to apoptosis and necroptosis, linked to many human diseases, and might be a good target for therapeutic intervention.

Human ARC contains 208 amino acids and is composed of two distinct domains, N-terminal caspase recruiting domain (CARD) domain and C-terminal P/E (proline and glutamic acid) rich domain (Fig. 1A). The CARD domain is a protein interaction module belonging to the death domain superfamily that includes the death domain (DD), death effector domain (DED), and pyrin domain (PYD)^24^,^25^,^26^,^27^. ARC exerts its function through multiple protein-protein interactions with apoptosis signaling molecules on both intrinsic and extrinsic pathways^28^,^29^. ARC is a unique apoptosis inhibitor in that it can antagonize both the intrinsic and extrinsic pathways. ARC inhibits the extrinsic pathway by interfering with DISC formation. ARC CARD interacts directly with the death domains (DD) of Fas and FADD, as well as with the death effector domains (DEDs) of procaspase-8^29^. Two different mechanisms for inhibition of the intrinsic pathway by ARC have been reported, functional inhibition of pro-apoptotic BAD, PUMA, and BAX through direct interaction between the ARC CARD and the C-terminus of BAX and the BH3 motif of BAD and PUMA,^30^ and functional inhibition of p53 via direct interaction between the ARC P/E domain and oligomerization domain of p53^31^. ARC also mediates crosstalk between intrinsic and extrinsic apoptosis pathways by sequestering Ca^2+^ in cytosol^32^. A significant amount of Ca^2+^ can bind to the P/E domain of ARC, and Ca^2+^ depletion by ARC can block the apoptosis process^32^. Interestingly, it has been reported that ARC forms a homo-dimer via the CARD domain and that dimerization abolishes the inhibitory activity of ARC.^29^

To better understand the CARD domain-mediated inhibition mechanism of ARC during apoptosis signaling, we report the first crystal structure of the CARD domain of ARC containing amino acids 1–95 at a resolution of 2.4 Å. Our structure was a dimer and revealed novel homo-dimerization interfaces, which might be critical for the inhibitory function. In addition, the high resolution structure revealed similarities and differences with those of other CARD domains that may be functionally relevant. Interestingly, ARC did not exhibit a typical death domain fold (the six helix bundle fold; H1–H6), and instead contained a five-helix bundle fold. H6 was not detected in the structure of ARC, indicating that it is dispensable for the function of the death domain superfamily.

## Results and Discussion

### Crystal structure of ARC CARD

**Figure 1:**
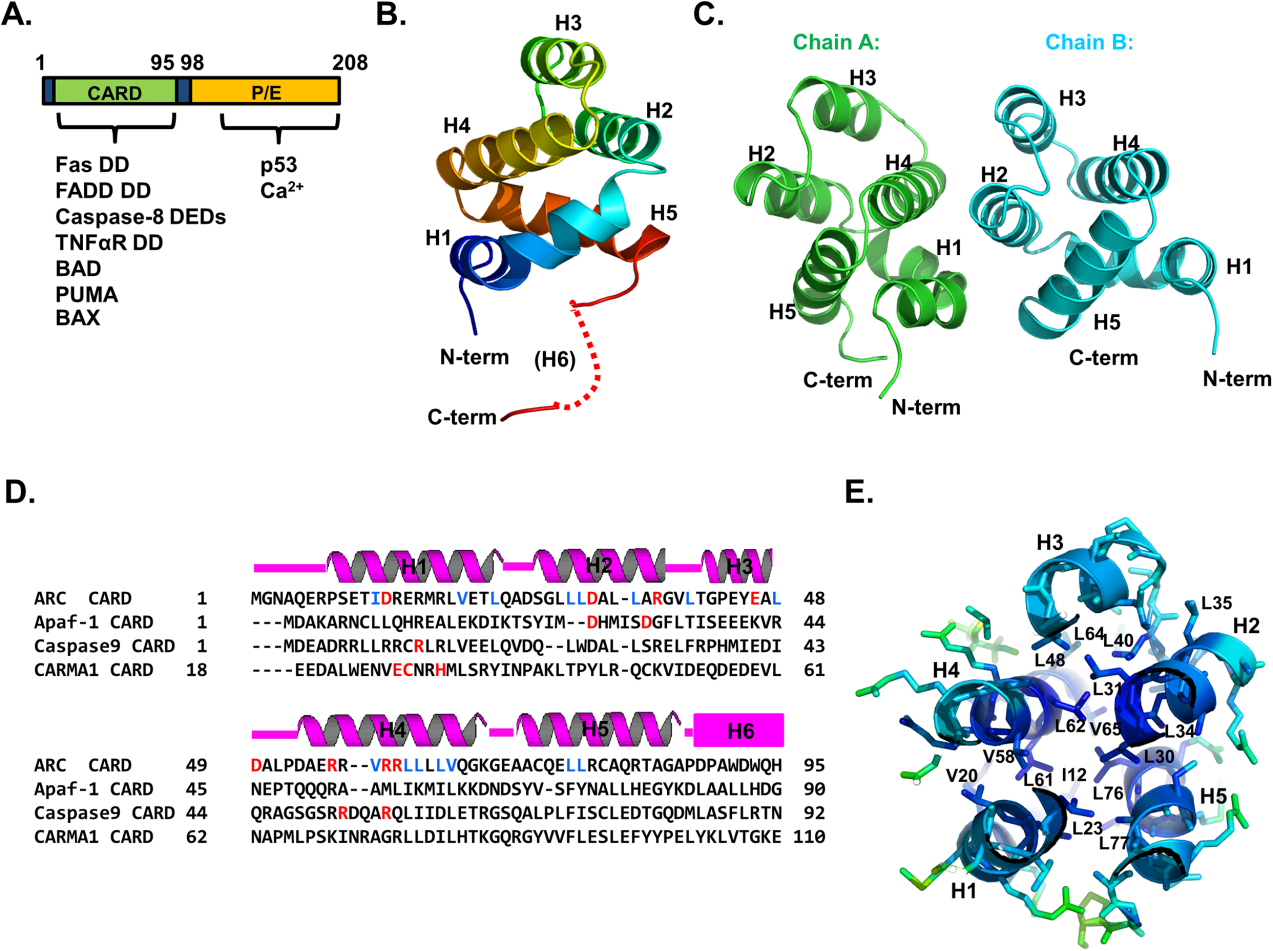
Crystal structure of ARC CARD. A. Domain organization and known binding proteins. CARD: caspase recruiting domain. P/E: proline and glutamine rich domain. DD: death domain, DED: death effector domain. B. Ribbon diagram of ARC CARD. The chain from the N- to C-termini is coloured from blue to red. Helices are labelled. The missing H6 at the c-terminus is shown as a red dashed line. C. Two ARC CARD molecules in the asymmetric unit. Chain A and Chain B are shown separately. D. Structure-based sequence alignment of ARC CARD with other CARD domains. Secondary structures (helices H1 to H5) are shown above the sequences. Residues at the hydrophobic core of ARC CARD are blue. Critical residues for the interaction with homo- or hetero- CARD molecules are red. Putative H6 is indicated by a pink box. E. Conserved central hydrophobic core. Hydrophobic residues essential to the formation of this core that stabilize H1 to H6 are shown as blue sticks and labelled.

ARC inhibits both the intrinsic and extrinsic apoptosis pathways via interaction with various apoptosis related proteins, including Fas, FADD, and caspase-8 (Fig. 1A). The ARC CARD domain is known to interfere with DISC formation through direct interaction with Fas DD, FADD DD, and caspase-8 DEDs (Fig. 1A)^29^.

The 2.4 Å crystal structure of the CARD domain of ARC (ARC CARD) was solved using a single-wavelength anomalous diffraction (SAD) method and refined to an R_work_ of 17.0% and an R_free_ of 20.0%. The high resolution structure of ARC CARD showed that it comprises five helices, H1 to H5, which is not the typical fold of the DD superfamily (Fig. 1B). Interestingly, the sixth helix (H6) was not detected at the typical location, indicating that H6 might be flexible in the CARD domain of ARC or adopt a loop structure instead. There were two monomers in the asymmetric unit, chain A and chain B (Fig. 1C). The model of chain A was built from residue 5 to residue 87 and residue 91 to residue 93, while that of chain B was built from residue 5 to residue 84. Putative H6 (from residue 88 to 93) could not be modelled due to the poor electron density map (Fig. 1B). Each monomer was almost identical and superimposed to an RMSD of 0.5 Å (Supplemental Fig. 1). Although an extraordinary form of H6 of the CARD domain was reported with a NOD1 CARD structure, this is the first report showing that H6 does not exist in the CARD domain. The amino acid sequence of the H6 region of ARC CARD is not a typical helix sequence when compared with those of other CARDs (Fig. 1D). The putative H6 region of ARC CARD (PDPAWDWQH) was predicted to be a random coil loop based on the secondary structure prediction program, indicating that H6 does not exist in ARC CARD and was instead replaced by a loop. This atypical structure of the CARD domain is the first case observed among the death domain superfamily. The length of H3 was shorter than that of other helices, which is a common characteristic of the death domain superfamily. The N and C termini of ARC CARD reside at the same side of the molecule. The helix bundle from H1 to H5 was tightly packed by a central hydrophobic core composed of I12, V20, L23, L30, L31, L34, L35, L40, L48, V58, L61, L62, L64, V65, L76, and L77 (Fig. 1E). Residues buried within the core forming the hydrophobic cluster of ARC CARD are not well conserved, but are the most abundant among different CARDs, indicating that ARC CARD might be one of the most compact and stable proteins among the death domain superfamily (Fig. 1D). The highly compact and ordered feature of ARC CARD is shown by the low average B factor of 57.9 Å^2^(Table 1).

The structural rigidity of the core part of ARC CARD and tentatively disordered H6 region of ARC CARD are distinct features that might be critical to the functions of ARC, which are mediated by various interacting proteins including the TNF receptor, FADD, BAD, and BAX. ARC CARD is the only member of the death domain superfamily that can interact with different subfamilies within the superfamily as well as with Bcl-2 family proteins. The relationship between the capacity of ARC to accommodate various binding partners and disordered H6 with a rigid core of ARC CARD should be investigated in future studies.

### Dimer interface within ARC CARD

**Figure 2:**
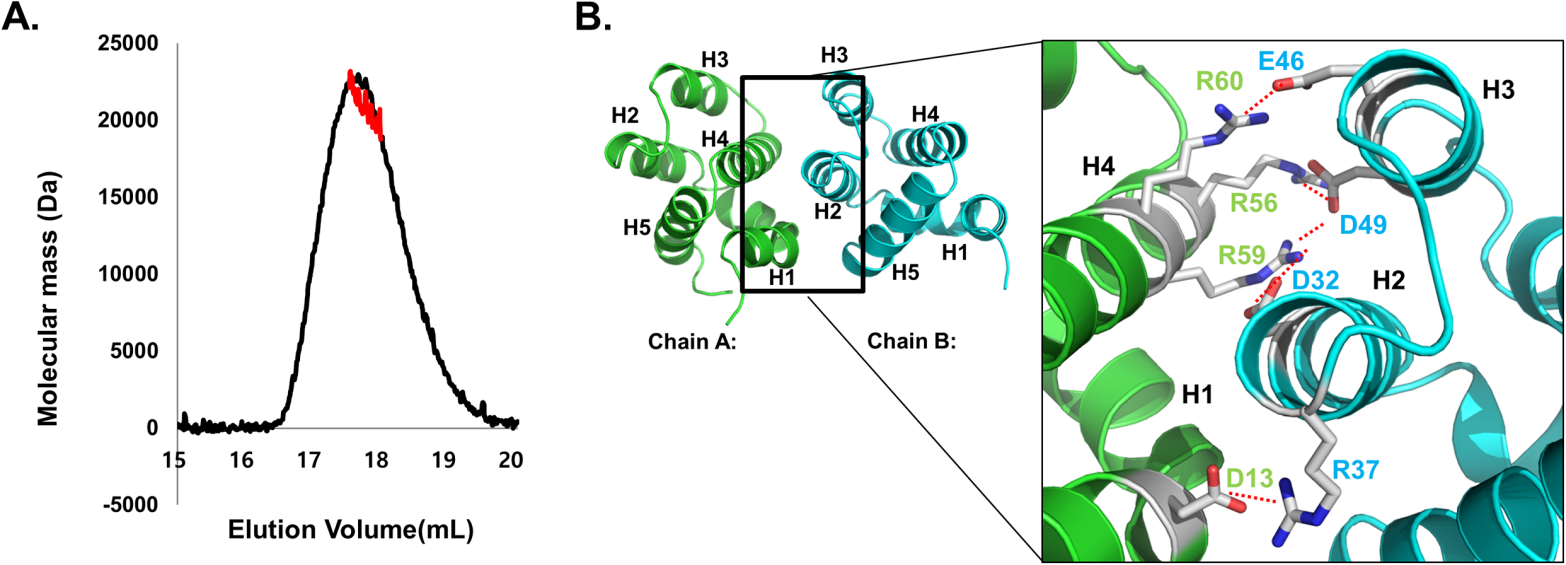
Dimeric interface of the structure of ARC CARD. A. Multi-angle light scattering (MALS) profile. The red line indicates the experimental molecular weight. B. Close-up view of the interacting residues in the interface between two monomers. Helices are labelled and residues involved in the contact are shown as sticks. Salt bridges are shown as dashed lines.

Size-exclusion chromatography and multi-angle light scattering (MALS) indicated that the isolated ARC CARD behaves as a dimer in solution (Fig. 2A). The calculated monomeric molecular weight of ARC CARD including the C-terminal His-tag was 11.763 Da, and the calculated molecular weight from MALS was 21.901 Da (0.9 % fitting error), with a polydispersity of 1.000 (Fig. 2A). It has been suggested that ARC can form a homo-dimer via the CARD, and that CARD-mediated dimerization of ARC abolishes its anti-apoptotic potential^29^. Our crystal structure also supports the dimeric form of ARC CARD in solution. The dimerization of the death domain superfamily including CARD is not surprising because many studies have shown that the DD superfamily can be homo-dimerized in solution^24^,^33^,^34^. The homo-dimeric structure of ARC CARD provides interesting insight into the homo-dimeric interfaces. The two ARC CARDs in the asymmetric unit are packed as an asymmetric dimer with an interface composed primarily of an electrostatic interaction (Fig. 2B). The total dimer surface buries 916 Å^2^ (a monomer surface area of 458 Å^2^), which represents 9% of the dimer surface area. The principal interaction forces are salt bridges and hydrogen bonds, which are made by D13 (on H1), R56 (on H4), R59 (on H4), and R60 (H4) from chain A and by D32 (on H2), R37 (on H2), E46 (on H3), and D49 (on H3) from chain B (Fig. 2B). At the peripheral region, D13 on H1 and R60 on H4 of chain A form salt bridges with R37 on H2 and E46 on H3 of chain B, respectively. In the central region of the interface, R56 and R59 on H4 of chain A form salt bridges with D49 on H3 of chain B. R59 of chain A also contributes to formation of a salt bridge with D32 on H2 of chain B. The interaction mode of the ARC homo-dimer is similar to that of the hetero-dimeric complex between caspase-9 CARD and APAF-1 CARD in that H1 and H4 of one CARD molecule interact with H2 and H3 of the other CARD molecule, primarily via charge-charge interactions^35^. This type of interaction belongs to the type I interaction among the three types of interactions detected in the death domain superfamily^14^,^25^. Although a unique disulfide bond-mediated homo-dimeric structure of the CARD domain including NOD1 and CARMA1 has been reported^33^,^34^, the current structure of ARC CARD was dimerized via a Type I interface, which is a novel method for the homo-dimeric CARD structure.

**Figure 3:**
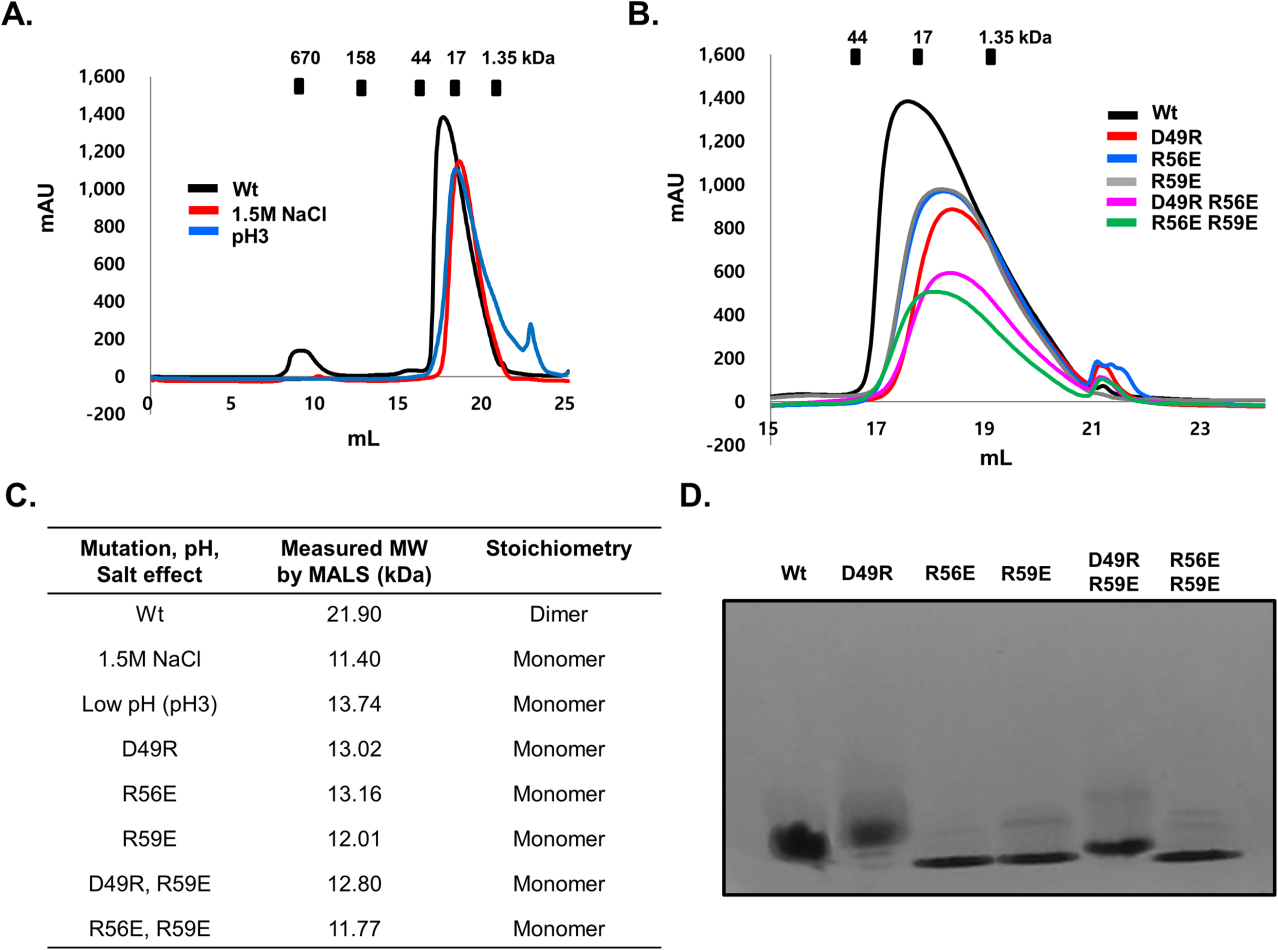
Analysis of the interface formed by homo-dimer of ARC CARD. A. Size exclusion chromatography profile of high salt and low pH condition. B. Size exclusion chromatography profile of various mutations. C. High salt, low pH, and mutation effects on dimer formation. Measured molecular weight and expected stoichiometry are shown. D. Native-PAGE. Wt indicates wild type.

To analyse the novel homo-dimeric structure of ARC CARD formed by large electrostatic interactions, we conducted size exclusion chromatography and MALS experiments in the presence of high salt (1.5 M NaCl) and low pH (pH 3), which resulted in the carboxylates being protonated and the charged interactions disrupted. As expected, ARC CARD became a monomer at high salt and low pH conditions (Fig. 3A and Supplemental Fig. 2A and 2B). Because D49, R56, and R59 are identified as critical residues for the formation of this novel interface, we also conducted a mutagenesis study to analyze the interface. Each residue (D49, R56 and R59) was mutated to opposite charges and used for the size exclusion chromatography and MALS experiments. As shown in Fig. 3B, the elution peak of the wild type observed upon size exclusion chromatography was moved to a monomeric place by mutation. The molecular weights determined by MALS and expected stoichiometry under various conditions are summarized in fig. 3C. The raw data for MALS are also shown in supplemental Fig. 2. The size exclusion chromatography and MALS experiment showed that the dimeric ARC CARD became a monomer via mutations of interface residue D49R, R56E, and R59E, and double mutants (Fig. 3B and 3C, and Supplemental Fig. 2). Changes in oligomerization in response to mutations were confirmed by native-PAGE. Down-shifting of the band by mutations indicates that dimeric ARC CARD became monomeric in solution in response to mutations (Fig. 3D). Generation of a smear up-shifting band by D49R mutation was an unexpected exception that we believe occurred in response to charge changes induced by mutagenesis. Because shifted or newly produced bands on native-PAGE are good indicators of the disruption or formation of the protein complex, the band generated by D49R may still be a monomer band. Another possibility is that D49R forms a dimer during concentration. At high concentration, weakly disrupted mutant D49R became a dimer in solution. Since the double mutant, D49R, R59E, generated a monomeric band (Fig. 3D), D49R might not be sufficient to disrupt charged interactions. To confirm that dimer disruption by mutations was not a result of unexpected structural changes caused by mutagenesis, but rather due to specific mutations that can disrupt the dimeric interface, we evaluated the far UV circular dichroic (CD) spectra (Supplemental Fig. S3). The CD spectra exhibited typical α-helical proteins, with two pronounced minima at 208 nm and 222 nm and a maxima at 195 nm, which is similar to that of the wild type and matched the molecular structure of other members of the DD superfamily well^36^. These results confirmed that disruption of ARC CARD dimerization is caused by specific mutagenesis, as expected, but not by the unexpected structural changes formed by mutagenesis.

Taken together, ARC CARD forms homo-dimer in solution, as expected, which was shown in a previous in vivo experiment^29^, and this dimerization is mediated by type I interaction with highly charged interactions detected in other death domain superfamily interactions and hetero-dimeric CARD domain interactions. Since this homo-dimerization of ARC CARD caused the loss of inhibitory activity of ARC, it is possible that ARC CARD uses this homo-dimeric interface to interact with other proteins. Revealing the protein interaction interface that was detected in our current study is essential to determination of the function of ARC.

### Comparison with other CARD structures

**Figure 4:**
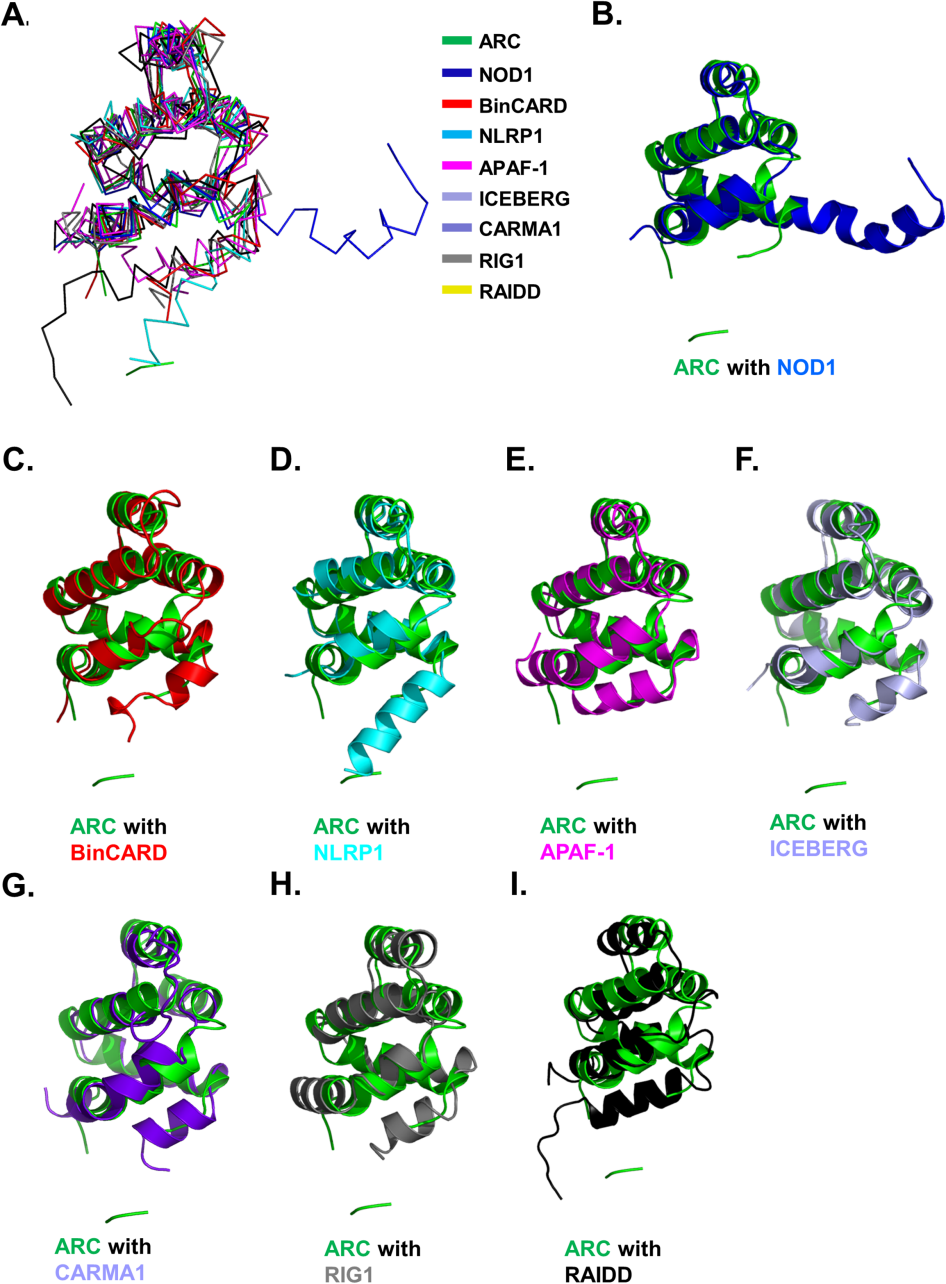
Superposition of ARC CARD with its structural homologues. A. ARC CARD (green colour) and eight structural homologues are superimposed. B-I. Pairwise structural comparison. ARC CARD is green and each counterpart is blue for NOD1 (B), red for BinCARD (C), cyan for NLRP1 (D), magenta for APAF-1 (E), light blue for ICEBERG (F), purple for CARMA1 (G), grey for RIG1 (H), and black for RAIDD (I).

A structural homology search with DALI^37^ showed that ARC CARD is highly similar to other CARDs. The top eight matches, which had Z-scores of 13.8 to 7.7, were NOD1, BinCARD, NLRP1, APAF-1, ICEBERG, CARMA1, RIG1, and RAIDD (Table 2). Superimposition of all nine CARDs indicated that the structures of all CARDs are well superimposed through H1 to H5, except for H6, which was not even detected in the structure of ARC CARD (Fig. 4A). H6 of NOD1 CARD is connected to H5 and not kinked. H6 of most CARDs is tightly packed in the central bundle, while H6 of NLRP1 CARD is located far from the central bundle (Fig. 4A).

Pair-wise structural alignments between ARC CARD and these other CARDs showed clear structural differences and similarities (Fig. 4B-G). The CARD best superimposed with ARC CARD was NOD1. Possession of atypical H6 was an interesting feature of ARC CARD and NOD1 CARD (Fig. 4B), while H1 of ARC CARD was longer than that of other CARDs. Based on the fragment residues of ARC CARD, the location of tentative H6 is similar to the location of H6 of NLRP1 (Fig. 4D). The conspicuous differences in H6 indicate the dynamic nature of H6 among various CARDs. The bent H1 detected in all of the CARD structures, including the current ARC CARD, is also an interesting feature that was only detected on the structure of CARDs.

ARC CARD has similar gross features in its electrostatic surface as other CARDs. For example, similar to the charged nature of most other CARDs, the ARC CARD surface is also composed of a mixture of positively and negatively charged features. The charged clusters are located in the interface of the homo-dimeric complex. Because CARDs are protein interaction modules, their surface features dictate their mode of interactions with partners. Based on analysis of the electrostatic surface of ARC CARD, it might be possible to use this charged interaction for homo or hetero dimeric complex formation.

### Conserved surface of ARC CARD: potential interaction site with Fas DD and FADD DD for inhibition of the DISC assembly

**Figure 5:**
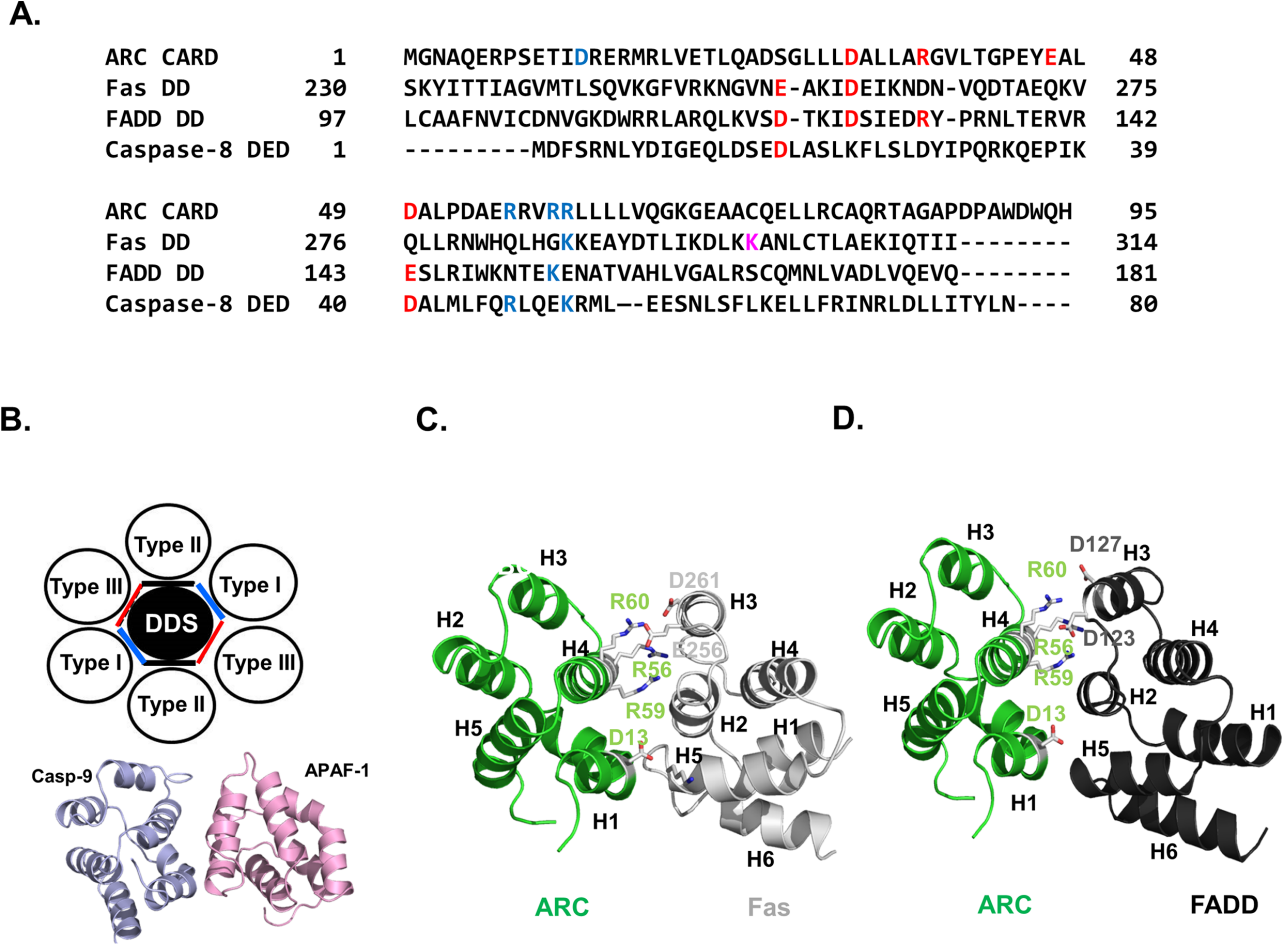
Model of molecular basis of inhibition of DISC formation by ARC CARD. A. Sequence alignment of ARC CARD with its binding partners, Fas DD, FADD DD, and caspase-8 DED. Residues critical to the homo-dimeric interaction of ARC CARD were blue for one side of the surface and red for the other side. Conserved residues on Fas DD, FADD DD, and caspase-8 DED are also shown as red or blue. B. The top panel shows a schematic diagram of the three types of interactions in the death domain superfamily complexes. DDS: death domain superfamily. The bottom panel shows the structure of the caspase-9 CARD (Casp-9)/APAF-1 complex, a representative CARD:CARD interaction formed by Type I interaction. C. Model of interaction between ARC CARD and Fas DD. Residues that might participate in the interaction are shown as sticks. D. Model of interaction between ARC CARD and FADD DD. Residues that might participate in the interaction are shown as sticks.

A number of residues on the surface of the ARC CARD that have been identified as critical to formation of the interface of the homo-dimeric complex are conserved on Fas (DD), FADD (DED), and caspase-8 (the first DED), which are known binding partners of ARC (Fig. 5A). These include D32, R37, D49, R56, R59, and R60 (Fig. 2B and 5A). D32 is conserved at both Fas DD and FADD DD, while D49 is conserved at FADD DD and caspase-8 DED1 and R60 is conserved at Fas DD and caspase-8 DED1. R37 and R59 are only conserved at FADD DD, while R56 is only conserved at caspase-8 DED1. D13 and E46, which are critical residues for formation of the homo-dimeric complex of ARC CARD, are not conserved at all (Fig. 5A).

Three types of interactions (types I, II, and III) at six unique interfaces (types Ia, Ib, IIa, IIb, IIIa, and IIIb) have been identified in the interactions with the death domain superfamily (Fig. 5B)^14^,^38^. The typical heterodimeric interaction with the CARD domain was identified by the crystal structure of caspase-9 CARD and APAF-1 CARD complex, and the interaction was formed by the type I interactions^35^. The Type Ia surface is primarily formed by residues at the H1 and H4 helices and interacts with the Type Ib surface, which is mainly formed by residues at the H2 and H3 helices (Fig. 5B).

Based on the sequence alignment, previously solved CARD complex structure, and location of the conserved residues critical to formation of the homo-dimeric complex of ARC CARD, we developed a tentative inhibitory model of ARC CARD by interacting with Fas DD or FADD DD. Because D13, R56, R59, and R60, which are critical residues for the formation of one side of the homo-dimeric interface of ARC CARD, are not relatively conserved at the Fas DD and FADD DD, this side of ARC CARD was used as a tentative interaction site. Fas DD or FADD DD was then superimposed on the ARC CARD located on the other side to make a Type I interaction. The ARC:Fas complex model showed that R56, R59, and R60 from ARC CARD form a massive charged interaction with D261 and E256 from Fas DD (Fig. 5C). D13 of ARC CARD might also be involved in the interaction by forming a salt bridge with K301 of Fas DD. In the case of the ARC:FADD complex model, D123 and D127 from H3 of FADD DD might have participated in the interaction with the basic patch (formed by R56, R59, and R60) of ARC CARD (Fig. 5D). The interfaces of the model structures were structurally and energetically favourable according to the PISA interface analysis program^39^.

The first homo-dimeric CARD structure of ARC and follow-up studies suggest an ARC-mediated molecular inhibitory mechanism of the DISC assembly, which is the critical signalling molecular complex in the extrinsic apoptosis signalling pathway. Since the multimeric CARD domain complex of RIG-1 and MAVS, which use all three types of interactions that have been detected for assembly of the death domain subfamily, were recently reported, the fact that ARC CARD forms a higher oligomeric complex with Fas DD or FADD DD during the inhibition process cannot be ignored. In this case, an extensive surface of ARC CARD with residues spreading through all five helices of its structure might have participated in assembly of the inhibitory complex. Further efforts to solve the complex structure are needed to obtain clearer insight into the molecular basis of inhibition by ARC. However, it has been reported that homo-dimerization of ARC CARD caused loss of inhibitory activity of ARC, indicating that our proposed model of the complexes with Fas DD and FADD is accurate. Revealing the protein interaction interface detected in the current study might be critical to the interaction with other binding partners for the proper inhibitory function of ARC during apoptosis and necroptosis.

ARC is prominently expressed in cardiac and skeletal muscle cells and prevents apoptotic cell death of those post-mitotic muscle cells. Involvement of ARC in cardiomyopathy under stress conditions was observed in a mouse model^40^, indicating that ARC might be tightly linked to heart diseases in humans. Inhibitory function of neuronal cell death of ARC was also reported in a mouse study^41^, indicating that ARC might be critical to neurodegenerative diseases in humans. High expression of ARC is frequently observed in malignant tumours, indicating that it might be essential to tumour development and progression. In this aspect, ARC could be a critical molecule that can control apoptotic and necrotic cell death; accordingly, failure to control the function of ARC leads to fatal disease in humans. Therefore, ARC might be a good target for therapeutic intervention, and the ARC CARD structure presented in this study might be the first step to understanding ARC CARD-mediated protein interactions and its inhibitory capacity. Small molecules that can control the activity of ARC by targeting the homo- and hetero- oligomeric interface might be good regulators of apoptotic and necrotic cell death and therefore potential drug candidates.

## Methods

### Protein expression and purification

The expression and purification methods used in this study have been described in detail elsewhere. Briefly, ARC CARD domain (amino acids 1–95) was expressed in *E. coli* BL21 (DE 3) under overnight induction at 20°C. The protein contained a carboxyl terminal His-tag and was purified by nickel affinity and size exclusion chromatography using a size 200 exclusion column 10/30 (GE healthcare) that had been pre-equilibrated with a solution of 20 mM Tris-HCl at pH 8.0 and 150 mM NaCl. The protein was then concentrated to 8-9 mg/ml for the crystallization trial.

### Crystallization and data collection

The crystallization conditions were initially screened at 20°C by the hanging drop vapour-diffusion method. The final diffractable crystals were grown on plates by equilibrating a mixture containing 1 μl of protein solution (8–9 mg ml^-1^ protein in 20 mM Tris-HCl at pH 8.0, 150 mM NaCl) and 1 μl of a reservoir solution containing 0.6 M ammonium phosphate and 0.1 M imidazole (pH 8.2) against 0.3 ml of reservoir solution. Selenomethionine-substituted ARC CARD was produced using a previously established method^42^ and crystallized similarly. A single-wavelength anomalous diffraction (SAD) data set was collected at the 5C beamline of the Pohang Accelerator Laboratory (PAL), Republic of Korea. Data processing and scaling were carried out using the HKL2000 package^43^.

### Structure determination and analysis

The single-wavelength anomalous diffraction (SAD) method was conducted using Phaser^44^. Model building and refinement were performed using COOT^45^ and Refmac5^46^, respectively. Water molecules were added automatically with the ARP/wARP function in Refmac5, then examined manually for reasonable hydrogen bonding possibilities. The quality of the model was checked with PROCHECK^47^. Ribbon diagrams and molecular surface representations were generated using the Pymol program^48^. Refinement statistics are summarized in Table 1.

### Sequence alignment

Amino acid sequences were analysed using Clustal W (http://www.ebi.ac.uk/Tools/clustalw2/index.html).

### Multi-angle light scattering (MALS)

The molar mass of the ARC CARD was determined by MALS. The target protein was injected onto a Superdex 200 HR 10/30 size exclusion column (GE Healthcare) that had been equilibrated in appropriate buffer. The chromatography system was coupled to a three-angle light scattering detector (mini-DAWN EOS) and a refractive index detector (Optilab DSP) (Wyatt Technology). Data were collected every 0.5 s at a flow rate of 0.2 ml/min and then analyzed using the ASTRA program, which gave the molar mass and mass distribution (polydispersity) of the sample.

### Mutagenesis

Site-directed mutagenesis was conducted using a Quick-change kit (Stratagene) according to the manufacturer's protocols. Mutagenesis was then confirmed by sequencing. Mutant proteins were prepared using the same method as described above.

### Native PAGE shift assay

Changes in oligomerization in response to mutations were monitored by native (non-denaturing) PAGE using a Phast System (GE Healthcare) with pre-made 8–25% acrylamide gradient gels (GE Healthcare). Separately purified proteins were pre-incubated at room temperature for 1 hour before loading the gel. Coomassie Brilliant Blue was used for staining and detection of shifted bands.

### Circular dichroism spectroscopy

The secondary structures were measured by circular dichroism (CD) spectroscopy using a J-715 spectropolarimeter at the Korea Basic Science Institute in South Korea. The spectra were obtained from 200 to 250 nm at 25°C in a 0.1 cm path length quartz cuvette using a bandwidth of 1.0 nm, a speed of 50 mm/min, and a 5s response time. Four scans were accumulated and averaged, after which the α-helical content was calculated from the molar ellipticity at 222 nm^49^.

## Additional Information

**How to cite this article**: Jang, T.-h. *et al*. Crystal structure of caspase recruiting domain (CARD) of apoptosis repressor with CARD (ARC) and its implication in inhibition of apoptosis. *Sci. Rep.* **5**, 9847; DOI:10.1038/srep09847 (2015).

**Accession Codes**: Coordinates and structural factors have been deposited in the RCSB Protein Data Bank. The PDB ID code is 4UZ0.

## Supplementary Material

Supplementary Information

## Figures and Tables

**Table 1:** Data collection and refinement statistics

Data set	Wild type
X-ray sourceSpace groupWavelength (Å)Resolution (Å)Total reflectionsUnique reflectionsAverage I/σ(I)R_merge_^a^RedundancyCompleteness (%)^b^	5C (SBII) at PAL*P6_5_*0.9776050.0–2.40 (2.45–2.40)88,22515,41245.9 (5.7)0.25 (0.56)7.2 (7.5)99.6 (100)
Refinement	
Resolution range (Å)No. of reflections of working setNo. of reflections of test setNo. of amino acid residues	27.4–2.424,77411,2591,312
No. of water molecules	20
R_cryst_ bR_free_ cR.M.S. bond length (Å)R.M.S. bond angle (°)Average B value (Å^2^) (protein)	0.170.200.0081.01057.9
Average B value (Å^2^) (solvent)	69.7
Ramachandran plot	
Most favored (%)	99.8
Allowed (%)	0.2

^a^R_cryst = _ ∑|<I>−I|/∑<I>.

^b^R_cryst = _ ∑| |Fo|−|Fc| |/∑|Fo|.

^c^R_free_ calculated with 5% of all reflections excluded from refinement stages using high-resolution data.Values in parentheses refer to the highest resolution shells.

**Table 2:** Structural similarity search using DALI

Proteins (accession numbers)	Z-score	RMSD (Å)	Identity (%)	References
NOD1 CARD (2nsn)	13.8	1.9	28	33
BinCARD CARD (4dwn)	12.6	1.7	20	50
NLRP1 CARD (4ifp)	11.7	2.2	22	51
APAF-1 CARD (2ygs)	11.5	1.8	15	35
ICEBERG CARD (1dgn)	10.6	1.7	22	52
CARMA1 CARD (4i16)	10.4	2.2	17	53
RIG1 CARD (4nqk)	9.1	2.2	10	54
RAIDD CARD (3crd)	7.7	3.0	22	55
