# Corrigendum: Crystal structure of caspase recruiting domain (CARD) of apoptosis repressor with CARD (ARC) and its implication in inhibition of apoptosis

**DOI:** 10.1038/srep17114

**Published:** 2015-12-18

**Authors:** Tae-ho Jang, Seong Hyun Kim, Jae-Hee Jeong, Sunghwan Kim, Yeon-Gil Kim, Hyun Ho Park

Scientific Reports 5: Article number: 9847; 10.1038/srep09847 published online: 06032015; updated: 12182015.

The original version of this Article contained a typographical error in the spelling of the author Yeon-Gil Kim which was incorrectly given as Yeun Gil Kim.

This has now been corrected in the PDF and HTML versions of the Article.

