# Supplementary material for: Crystal structure of caspase recruiting domain (CARD) of apoptosis repressor with CARD (ARC) and its implication in inhibition of apoptosis
Source: Sci Rep. 2015 Jun 3;5:9847. doi: 10.1038/srep09847 (PMC4453921; doi:10.1038/srep09847)
Supplement: Supplementary Information [file srep09847-s1.pdf]

MICROBIOLOGY, BIOCHEMISTRY, STRUCTURAL BIOLOGY

Hyun Ho Park, Phone. +82-53-810-3045. Fax: +82-53-810-4516. E-mail: hyunho@ynu.ac.kr

# Crystal structure of caspase recruiting domain (CARD) of apoptosis repressor with CARD (ARC) and its implication in inhibition of apoptosis

Tae-ho Jang[1*], Seong Hyun Kim[1*], Jae-Hee Jeong[2*], Sunghwan Kim[3], Yeun Gil Kim[2#] & Hyun Ho Park[1#]

[1]Department of Biochemistry, Yeungnam University, Gyeongsan, 712-749, South Korea,

[2]Pohang Accelerator Laboratory, Pohang University of Science and Technology, Pohang, 790-784, South Korea,

[3]New Drug Development Center, Daegu-Gyungpook Medical Innovation Foundation, Daegu, 701-310, South Korea.

Supplementary Figure S1

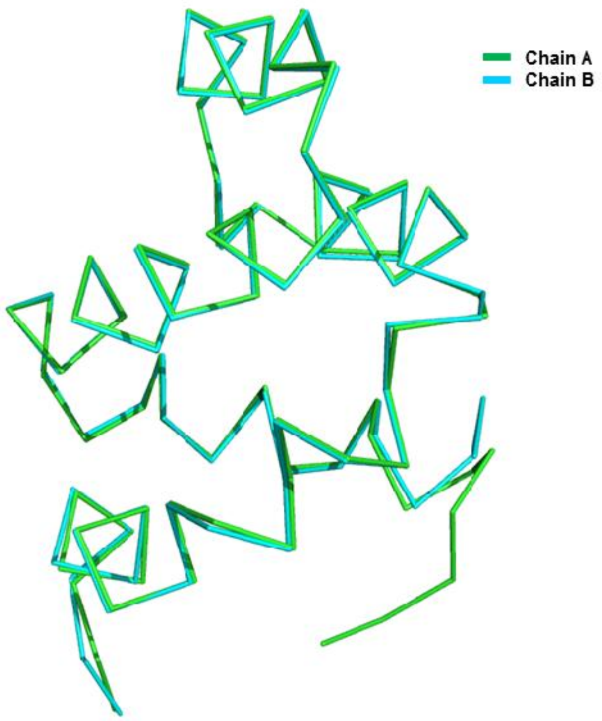


Figure S1. Structural comparison between Chain A and Chain B. Chain A and Chain B were superimposed.

Supplementary Figure S2

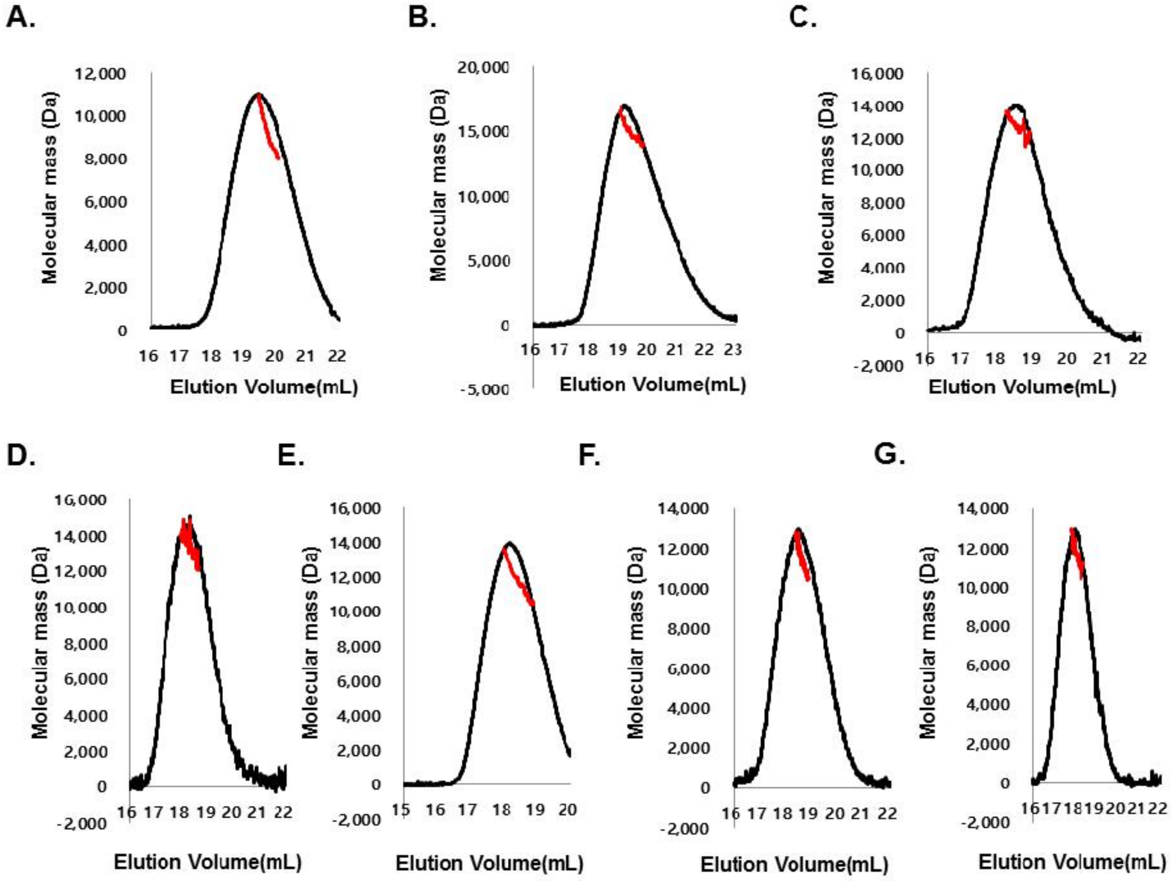


Figure S2. MALS results. The red line indicates the experimental molecular mass. A. MALS result from high salt condition. Buffer used for size exclusion chromatography followed by MALS experiment contains 20 mM Tris-HCl pH 8.0 with 1.5 M NaCl. B. MALS resuls from low pH condition. Buffer used for size exclusion chromatography followed by MALS experiment contains 20 mM sodium citrate pH 3.0 with 150 mM NaCl. C. MALS result from D49R. D. MALS result from R56E. E. MALS result from R59E. F. MALS result from D49R,R59E double mutant. G. MALS result from R56E,R59E double mutant. Buffer used for C~G experiments contains 20 mM Tris HCl pH 8.0 with 150 mM NaCl.

Supplementary Figure S3

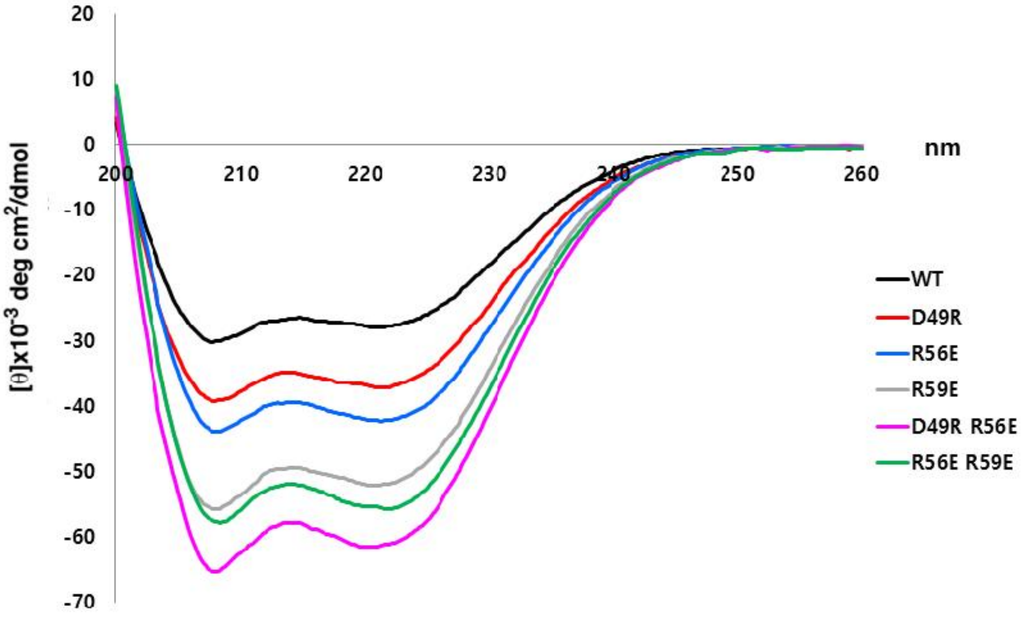


Figure S3. Circular dichroic spectra of purified wildtype (WT) and five mutants.
